# On the Traceability of Honey by Means of Lanthanide Distribution

**DOI:** 10.3390/foods12091803

**Published:** 2023-04-26

**Authors:** Federica Gulino, Elisa Calà, Christian Cozzani, Lorenzo Vaccari, Matteo Oddone, Maurizio Aceto

**Affiliations:** 1 Dipartimento per lo Sviluppo Sostenibile e la Transizione Ecologica, Università degli Studi del Piemonte Orientale, Piazza S. Eusebio, 5, 13100 Vercelli, VC, Italy; federica.gulino@uniupo.it (F.G.); elisa.cala@uniupo.it (E.C.);; 2 Thermo Fisher Scientific, Strada Rivoltana, 20090 Rodano, MI, Italy; matteo.oddone@thermofisher.com

**Keywords:** honey, lanthanides, ICP-MS, ICP-OES, traceability, chain, trace elements

## Abstract

Honey is a natural food appreciated all over the world since antiquity due to its well-recognised beneficial properties. However, it is also considered among the most counterfeited foods. Therefore, analytical methods are currently being developed to allow the verifying of its geographic provenance and its botanical origin. Trace- and ultra-trace elements are usually exploited as chemical descriptors in authentication studies, as they allow the properties declared in the label to be verified. A different matter is to trace a food by means of traceability, that is, to find the link between a food and the soil in which this food originates. For traceability, it has been demonstrated in several studies that the lanthanides are particularly useful to find this link. In the present study, the traceability of the honey chain has been studied by means of ICP-MS and ICP-OES analysis, by comparing the lanthanide distributions of 17 different monofloral honey chains, each one composed of honey, flowers and soil in which such flowers grew. The results show that, while the fingerprint of soil, described by the lanthanide distribution, is transmitted unaltered from soil to flowers, a slight fractionation on the heavier lanthanides (from Dy to Lu) occurs in the passage from flowers to honey.

## 1. Introduction

The origin of food is subjected to wide controls by government institutions. It regards both the aspects of health protection and fraud prevention. It is therefore important to develop analytical methods for this task that are both sensitive and reliable.

Honey is a natural food appreciated since antiquity. Two types can be distinguished: one produced by honeybees (*Apis mellifera*) from carbohydrates-containing plant exudates (nectar honey), another derived from sugar-rich excretions left by insects of the *Hemiptera* order (*Hemiptera*) that suck fluids from plants (honeydew honey). The first is the most common honey, while the second type is mainly produced in areas where flowering is poor, such as mountain coniferous forests [1].

The organoleptic and physical properties of honey, such as colour, aroma and flavour, depend on the kind of flowers, which in turn depend on their geographical origin.

Moreover, most of the honey sold on the market comes from a blend of different flowers and the result is the multifloral honey, while if a single type of flower predominates, the honey produced is termed monofloral honey, and this makes its commercial value higher because it has particular appreciated organoleptic properties.

Some international regulations, such as the Codex Alimentarius of the United Nations [2] and EU regulation 110/EC/2001 [3], protect the production and composition of honey. Moreover, the botanical type and origin of the product must be declared on the label. Despite the label information, which should protect the consumers from commercial frauds, honey is one of the five foods most subjected to counterfeit [4].

Despite the complexity of honey, it is possible to verify the botanical origin and different validated methods are available, such as elemental analysis [5,6], sensory analysis [7,8], melissopalynology (percentage of the different categories of pollen grains in honey, determined by microscopy) [9,10], near-infrared spectroscopy [11,12], FT-IR spectroscopy [13,14], Raman spectroscopy [10,15], potentiometric electronic tongue [16], head-space flash gas chromatography [1] and other physicochemical analyses (e.g., electrical conductivity, pH, total acidity and water activity) [17]. The methods for authentication of honey have been recently reviewed [18,19,20,21,22].

A harder task, however, is to recognise the geographical origin of a honey, for which the knowledge of the botanical type is not informative enough. For this purpose, good chemical descriptors in the food sector are the microelements or elements at trace- and ultra-trace concentrations [23,24]. Several works have demonstrated the usefulness of microelements in determining the geographic origin of honey [25,26,27].

A different approach is studying the analytical traceability of a food, that is, finding a link between its composition and that of the soil from which such food originates. While most studies exploit chemical descriptors to assess the authenticity of foods, i.e., to verify the properties reported in the label, very few of them study the traceability. Oddone et al. [28] verified the link between hazelnuts and the soil in which hazel trees grow, finding that the fingerprint given by the lanthanide distribution was passed unaltered from soil to fruits. This study suggested that the geochemical properties of lanthanides make them particularly suitable for the traceability of food. Similar conclusions were drawn from other works of the same authors [29,30,31,32].

The focus of this work is to assess whether the distribution of microelements, and in particular of the lanthanides, determined by means ICP-MS and ICP-OES spectrometry, can be useful for verifying the provenance of honey and therefore determining its geographical origin. The traceability study involves the analysis of (1) monofloral honey, (2) the flowers from which bees pick nectar and (3) the soil in which flowers grow.

Firstly, we made a critical evaluation of the honey production chain in order to verify whether the single passages could alter or not the original elemental composition. Honey is a complex product, without a real industrial production chain; in fact, the bees are the only crucial workers in its production. Worker bees collect nectar from plants that grow up to 3 km from the hive, inside an area of, on average, 7 km^2^ around the hive itself [7]. Then, they store the nectar in a second stomach shaped like a sac and, here, the nectar becomes mixed with enzymes that change their chemical composition and pH. The bee then returns to the hive to begin the regurgitation process, where the nectar is passed on from bee to bee until it is broken down into simple sugars and, after that, this partially digested nectar is deposited into honeycombs.

There are a few methods for removing honey from the hive, but all involve exclusively mechanical steps, so that external contributions could be excluded.

After collection from the hive, most of the honey sold is heated up between 49 °C and 74 °C; while some of the healthy properties of honey are lost during processing, it can be safely hypothesised that the initial elemental composition is not altered. Moreover, no external substances should be added to the packaged honey, which could influence artificially the natural elemental distribution. In this way, the honey elemental composition should be only driven by (1) the original distribution in soil, due to the orogenetic process, (2) the plant metabolism and (3) the process of digestion of bees.

The third passage can be considered the most critical. During the digestion process, bees mixed the nectar with specifically enzymes that break sugars down, transform the nectar chemical composition and pH, reduce water and bacteria and make the product more suitable for long-term storage. Then, a crucial point of this work will be to assess whether the nectar digestion process, which involves the addition of bee’s enzymes, can cause variation to the initial elemental composition.

The samples analysed in this study were all from Piemonte (Italy). Twenty-one different sets were collected, each one composed of soil, flowers and honey. The selection of the chains was carried out on the basis of the reliability of the beekeepers and of the size of the areas in which bees worked (each one being a few km^2^). The sets were the following:

- Alpignano (province of Turin):
  - Acacia;
  - Taraxacum;
  - Linden.
- Bagnasco (province of Cuneo):
  - Chestnut.
- Borgo d’Ale (province of Vercelli):
  - Acacia 2020;
  - Acacia 2021;
  - Chestnut 2020;
  - Chestnut 2021, pollen.
- Cocconato (province of Asti):
  - Acacia 2020;
  - Acacia 2021;
  - Linden.
- Colle del Lys (province of Turin):
  - Clover 2020;
  - Clover 2021.
- Giaveno (province of Turin):
  - Chestnut.
- Montiglio (province of Asti):
  - Acacia;
  - Linden.
- Parco Colletta (province of Turin):
  - Linden 2020;
  - Linden 2021.
- Robilante (province of Cuneo):
  - Chestnut/clover/taraxacum.
- Romano Canavese (province of Turin):
  - Chestnut.
- Settimo Vittone (province of Turin):
  - Clover.

Twenty of these sets were monofloral honeys, while the chain of Robilante was a multifloral honey produced by bees from chestnut, clover and taraxacum flowers. The chestnut honey chain of Borgo d’Ale also contains a sample of pollen.

## 2. Materials and Methods

### 2.1. Reagents

High-purity water (HPW) with resistance > 18 MΩ·cm was produced with a Milli-Q (Milford, MA, USA) apparatus. TraceSelect hydrogen peroxide 30%, nitric acid 69% and hydrochloric acid 37% were purchased from Fluka (Milan, Italy). Polypropylene and polystyrene vials, used, respectively, for sample storage and analysis with an autosampler system, were kept in 1% nitric acid and then rinsed with high-purity water when necessary. Elements stock solutions (Inorganic Ventures, Lakewood, NJ, USA) were used for external calibration and internal standardisation.

### 2.2. Sample Collection

Samples of soil, flowers and honey were collected from 11 beekeepers working in different areas of Piemonte (north-western Italy). Flowers and honey samples were from acacia (*Robinia pseudoacacia*) (n = 6), chestnut (*Castanea sativa*) (n = 5), clover (*Trifolium alexandrinum*, *Trifolium repens*, *Trifolium incarnatum*) (n = 3), linden (*Tilia cordata*, *Tilia platyphyllos*, *Tilia americana*) (n = 5) and taraxacum (*Taraxacum officinale*) (n = 1). One chain was obtained from mixed floral species. In some cases, we collected different monofloral honey samples from a single beekeeper that were produced by bees working in the same area. In other cases, we collected honey samples of the same monofloral variety produced in two different years. In a single case (chestnut honey), it was possible to achieve a sample of pollen. The total number of honey chains collected was 21. The botanical composition of the honeys has been determined by means of melissopalynological analysis.

Soil and flowers samples were taken inside a territory of 3 km near each hive, which is reputed to be, on average, the distance which bees naturally can reach [33].

### 2.3. Sample Treatment

#### 2.3.1. Soil

The analysis of soil samples was carried out following a standardised procedure: 1 kg was dried at 120 °C overnight, after which it was sieved (φ 0.2 mm); aliquots of ca. 1 g were taken, put in PTFE vessels and extracted with 2 mL of hydrogen peroxide 30% and 6 mL of aqua regia, then the vessels were put inside a Start D microwave oven system (Milestone, Sorisole, Italy). The heating treatment increased the temperature from 25 °C to 180 °C over 15 min and kept the temperature constant for 10 min. The resulting mixture was taken to 50 mL with HPW in a polypropylene tube, then centrifuged at 6000 rpm for 10 min and the supernatant was collected. Solutions were diluted 1:100 with HPW prior to ICP analysis.

#### 2.3.2. Flowers

Flower samples were dried in oven at 80 °C overnight and then, after grinding the dried samples, ca. 0.5 g were subjected to acid digestion in PTFE vessels with 2 mL of nitric acid 69%, 1 mL of hydrogen peroxide 30% and 5 mL of HPW, then the vessels were put inside the microwave oven system. The heating treatment increased the temperature from 25 °C to 180 °C in 15 min and kept the temperature constant for 10 min. The resulting solution was taken to 50 mL with HPW in a polypropylene tube. Solutions were then diluted 1:10 with HPW prior to ICP analysis.

The same procedure was applied to the sample of pollen from chestnut.

#### 2.3.3. Honey

Honey is mainly composed of sugar and water; therefore, two different methods were compared for its pre-treatment—acid digestion in microwave oven and dry ashing in microwave oven:

- Acid digestion was applied to 1.0 g of honey weighted directly into PTFE vessels, then 1 mL of 30% hydrogen peroxide, 2 mL of nitric acid 69% and 5 mL of HPW were added and the vessels were put inside the microwave oven system. The heating treatment increased the temperature from 25 °C to 180 °C in 15 min and kept the temperature constant for 10 min. The resulting solution was taken to 50 mL with HPW in a polypropylene tube. Solutions were then diluted 1:10 with HPW prior to ICP analysis.
- Dry ashing was applied by weighting ca. 15 g of honey into a porcelain capsule, then putting the capsule inside a Milestone (Sorisole, Italy) Pyro 260 microwave ashing system. The heating cycle was as follows: room temperature to 150 °C in 10’; hold at 150 °C for 20’; up to 500 °C in 20’; hold at 500 °C for 30’; up to 750 °C in 10’; hold at 750 °C for 30’; up to 1000 °C in 10’; hold at 1000 °C for 30’. The resulting ash was completely dissolved in 2.0 mL of ultrapure concentrated nitric acid and taken up to 50 mL with HPW in a polypropylene tube. Solutions were then diluted 1:10 with HPW prior to ICP analysis.

After comparing the results on a sample of honey, dry ashing was chosen as pre-treatment instead of acid digestion, because all the analytes resulted in being above the detection limits, while the same did not hold true for acid digestion. The reason is of course due to the higher amount of sample managed with dry ashing with respect to acid digestion (15 g vs. 1 g) that maximised the content of trace elements in the solutions to be analysed. One drawback of dry ashing was the partial or total loss of volatile elements (e.g., As, Cd, Hg, Pb) so that, for the determination of these specific elements, acid digestion was used instead.

### 2.4. ICP-MS Analysis

Determination of trace and ultra-trace elements was carried out with a Thermo Scientific (Waltham, MA, USA) iCAP^TM^ RQ inductively coupled plasma mass spectrometer with single quadrupole technology. The instrument is equipped with an ESI (Omaha, NE, USA) PFA 100 MicroFlow nebulizer, a Peltier-cooled quartz spray chamber operating at 3 °C, a 2.0 mm ID quartz injector and a demountable quartz torch. Measurements were carried out exploiting an ESI (Omaha, USA) SC-4 DX autosampler. To overcome spectral interferences, the Collision Cell Technology (CCT) was used with He gas at 3.5 mL/min and a kinetic energy discrimination (KED) barrier of 2 V; the CCT-KED device was particularly useful in minimising the interferences of oxides (e.g., ^141^Pr^16^O on ^157^Gd) in the determination of lanthanides. Sensitivity performances were comparable between standard and KED mode (Ce > 500.000 cps/ppb in both modes), thanks to the extraordinary efficiency of Qcell flatpole; therefore, only the KED experimental setting was used. Instrument and accessories were PC-controlled by Qtegra^TM^ v. 2.10.4345.136 software. Instrumental parameters were as follows: forward power, 1550 W; plasma gas flow, 14.0 L/min; nebulizer gas flow, 0.9 L/min; auxiliary gas flow, 0.8 L/min. Three replicates were made for a total acquisition time of 180 s. The following isotopes were used: ^9^Be, ^23^Na, ^24^Mg, ^27^Al, ^31^P, ^39^K, ^44^Ca, ^47^Ti, ^51^V, ^52^Cr, ^55^Mn, ^57^Fe, ^59^Co, ^60^Ni, ^63^Cu, ^66^Zn, ^75^As, ^78^Se, ^89^Y, ^90^Zr, ^95^Mo, ^111^Cd, ^121^Sb, ^137^Ba, ^139^La, ^140^Ce, ^141^Pr, ^146^Nd, ^147^Sm, ^153^Eu, ^157^Gd, ^159^Tb, ^163^Dy, ^165^Ho, ^166^Er, ^169^Tm, ^172^Yb, ^175^Lu, ^181^Ta, ^182^W, ^205^Tl, ^232^Th and ^238^U. ^103^Rh, ^115^In and ^193^Ir were used as internal standards.

Interference due to oxide formation was evaluated as follows: CeO^+^/Ce^+^ < 0.5% in KED mode. A stability test performed before each session by monitoring ^7^Li, ^59^Co, ^115^In, ^140^Ce and ^238^U yielded a precision higher than 2%. The instrumental precision was better than 2% for trace and ultra-trace elements, while the overall precision, involving both sample preparation and instrumental analysis, was better than 5%, as calculated on five genuine replicates. Background signals were monitored at 5, 101 and 220 *m*/*z* to perform a sensitivity test on the above-reported analyte masses. CCS-1, CCS-2, CCS-4, CCS-5 and CCS-6 multi-element standard solutions from Inorganic Ventures (Christiansburg, VA, USA) were used to prepare 100, 10, 1 and 0.1 µg/L solutions in 1% nitric acid. Internal standardisation monitoring ^103^Rh, ^115^In and ^193^Ir isotopes was used to correct for instrumental drifts by means of interpolation to yield a better correction; the three isotopes were added to all solutions analysed at 10 μg/L. Limits of detection (LOD) and limits of quantification (LOQ), calculated as 3 and 10 times the standard deviation of blank measurements, respectively, can be found in a previous publication [34].

### 2.5. ICP-OES Analysis

Determination of major and minor elements was carried out with a Spectro (SPECTRO Analytical Instruments GmbH, Kleve, Germany) Genesis ICP-OES simultaneous spectrometer with axial plasma observation. Instrumental parameters were as follows: pump speed, 2.0 mL/min; RF generator, 40 MHz; RF, 1300 W; plasma power, 1400 W; plasma gas outlet, 12 L/min; auxiliary gas flow rate, 0.90 L/min; nebulizer flow rate, 0.96 L/min. The elements determined were the following (in parentheses the wavelength of acquisition): Al (396.152 nm), B (249.773 nm), Ba (233.527 nm), Ca (317.993 nm), Co (228.616 nm), Cr (205.552 nm), Cu (324.754 nm), Fe (259.941 nm), K (766.491 nm), Li (670.780 nm), Mg (285.213 nm), Mn (257.611 nm), Na (589.592 nm), Ni (231.604 nm), P (177.495 nm), Rb (420.185 nm), S (180.731 nm), Si (251.612 nm), Sr (460.733 nm), Ti (336.121 nm), V (292.464 nm) and Zn (213.856 nm). CCS-4 and CCS-5 multi-element standard solutions from Inorganic Ventures (Christiansburg, VA, USA) were used to prepare 10, 5, 1, 0.5 and 0.1 mg/L solutions in 1% nitric acid. Limits of detection (LOD) and limits of quantification (LOQ), calculated as 3 and 10 times the standard deviation of blank measurements, respectively, can be found in a previous publication [34].

### 2.6. Analysis of Certified Samples

The performance of analytical protocols chosen was evaluated through the analysis of three certified standard materials. For soil, we used SRM 2586 (*Trace Elements in Soil Containing Lead from Paint*) certified material from NIST; for flower and honey samples, we chose a material with a prevailing organic matrix: SRM-1573A (*Tomato leaves*) from NIST. The results are reported in Table 1 and Table 2, expressed as mg/kg. The analyses carried out showed an acceptable agreement between certified and observed concentration values.

### 2.7. Data Analysis

For classification of samples, multivariate pattern recognition methods were used. Classification was carried out using XLSTAT (Addinsoft^TM^, Paris, France) v. 2012.2.02 software, running as add-on for Microsoft Excel 2010 (Microsoft Corporation, Redmond, WA, USA).

## 3. Results

The results obtained from ICP analysis of soil, flower and honey samples are resumed in Table 3, in which the minimum, maximum and average values are reported for every element determined in the three matrices.

### 3.1. Traceability of the Honey Chain

After pre-treatment of the samples and ICP analysis, the traceability of the honey production chain was evaluated by comparing the distributions of the elements determined in soil, flowers and the final product, that is, honey. The elemental distributions determined by means of ICP-OES (major and minor elements) and ICP-MS (trace- and ultra-trace elements) must be normalised; however, as the absolute concentrations are hardly comparable: in fact, the concentrations in soil are from 2 to 4 times higher than in honey. Data were therefore normalised with respect to cerium (Ce), as already performed in previous works by the same authors [28,29,30,31,32] according to the following algorithm:

(1) $$Element_{ij,norm}=\frac{Element_{ij}}{Ce_{j}}$$

In this equation, *Element_ij_* indicates the concentration of the *i*-th element in the *j*-th sample, *Element_ij,norm_* indicates the Ce-normalised concentration, while *Ce_j_* is the concentration of Ce in the *j*-th sample expressed in the same unit. In addition, data are presented in a logarithmic scale rather than in a linear scale in order to reduce the effect of some major elements, typically alkaline and alkaline-earth elements, which, once normalised, make the comparison less readable.

**Figure 1:**
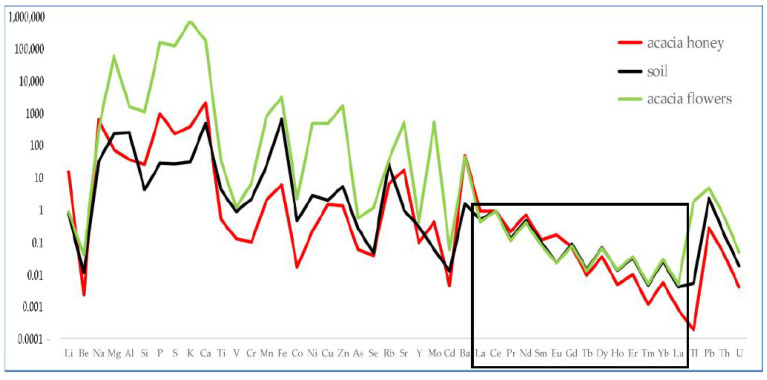
Distribution of elements in soil, acacia flowers and acacia honey produced at the Alpignano site. Data were normalised to Ce.

Figure 1 shows the Ce-normalised distribution of all the elements determined in an acacia honey chain from the site of Alpignano. In the insert rectangle, the group of lanthanides is highlighted.

It is possible to observe that the lanthanides have a completely different behaviour with respect to the other elements: in fact, their distribution seems to be relatively unaltered in the passage from soil to honey; therefore, they act as a fingerprint of soil. We can assume that they represent the behaviour of elements passively assumed by plants. On the contrary, potentially toxic elements such as beryllium (Be), cadmium (Cd), lead (Pb), thallium (Tl) are lower in honey than in both soil and flowers, as they are probably excluded by the metabolism of bees.

**Figure 2:**
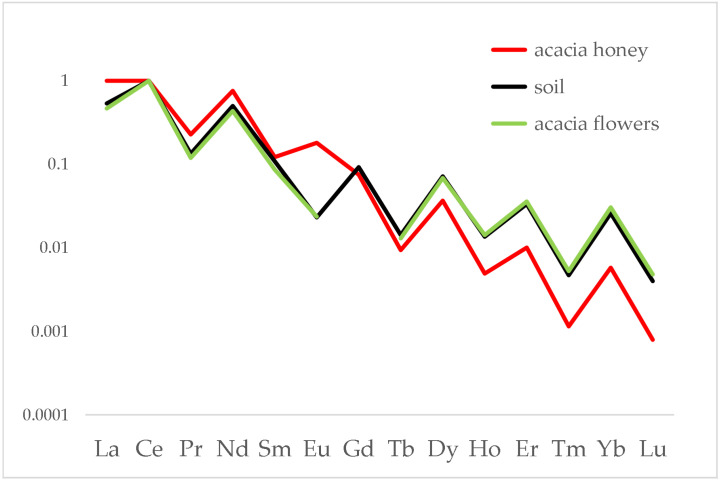
Distribution of lanthanides in soil, acacia flowers and acacia honey produced at the Alpignano site. Data were normalised to Ce.

To evaluate the role of lanthanides, Figure 2 reports an enlarged view on this group of elements alone.

The enlarged view on the lanthanide distribution allows their behaviour in this chain to be better understood. First of all, the distributions of soil, flower and honey follow strictly the Oddo–Harkins rule [35]. However, while the distributions in soil and flowers are perfectly overlapped, as expected according to previous studies [36,37,38], the distribution in honey is only partially overlapped because it shows fractionation on the heavier lanthanides, i.e., from dysprosium (Dy) to lutetium (Lu).

A different behaviour was registered for europium (Eu), which seems to be higher than expected. This can be explained, indeed, in terms of a positive interference from barium (Ba): due to the low-resolution power of the quadrupole used, it was not possible to exclude the interference of ^16^O^135^Ba^+^ and ^16^O^137^Ba^+^ on, respectively, ^151^Eu and ^153^Eu. Therefore, the measurements of Eu reflect basically the content of Ba in honey, which is more than 100 times higher than Eu; Ba, however, does not follow the behaviour of the lanthanides, being an ion vicariant of calcium (Ca) and therefore assumed actively by bees.

**Figure 3:**
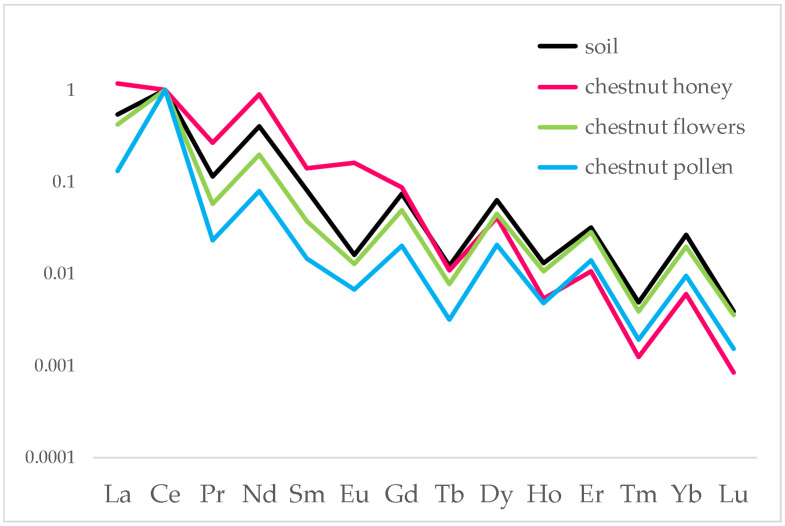
Distribution of lanthanides in soil, chestnut flowers, chestnut honey and pollen produced at the Borgo d’Ale site. Data were normalised to Ce.

In the case of the chestnut honey chain of Borgo d’Ale, a sample of pollen was also available. Pollen was analysed following the same pre-treatment method of flowers. Figure 3 reports the results of this chain.

The behaviour of flowers and honey is similar to all other chains, with the first overlapping the distribution of soil and the second showing fractionation on the heavier lanthanides. Pollen shows a more fractionated behaviour: this can be justified considering that, in the hive, pollen is processed by bees to prevent its bacterial putrefaction.

### 3.2. Different Floral Species on the Same Soil

**Figure 4:**
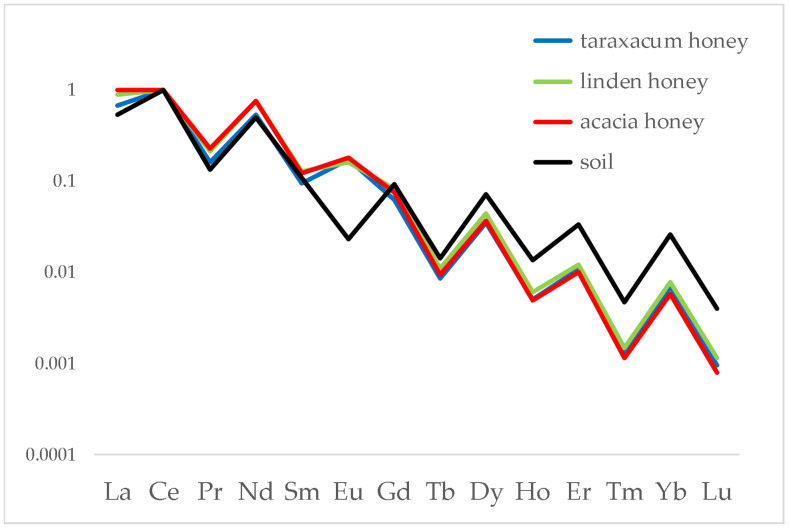
Distribution of lanthanides in acacia, linden and taraxacum honeys and in the relative soil. Data were normalised to Ce.

To verify whether the fingerprint of soil is—at least partially—transmitted to honey regardless of the floral species, we compared the distributions of monofloral honeys obtained from different species grown on the same territory. Figure 4 shows a comparison of honey samples from acacia, linden and taraxacum obtained from bees that collected nectars from flowers growing the same area, that of Alpignano.

The results show clearly that the three honeys have the same lanthanide distribution, with heavy lanthanides showing the same fractionation behaviour with respect of soil. A similar behaviour has been noted on other honey chains among those studied in this work. It is therefore demonstrated that the fingerprint of soil is transmitted to honey, regardless of the botanic variety.

### 3.3. Different Floral Species in the Same Honey

**Figure 5:**
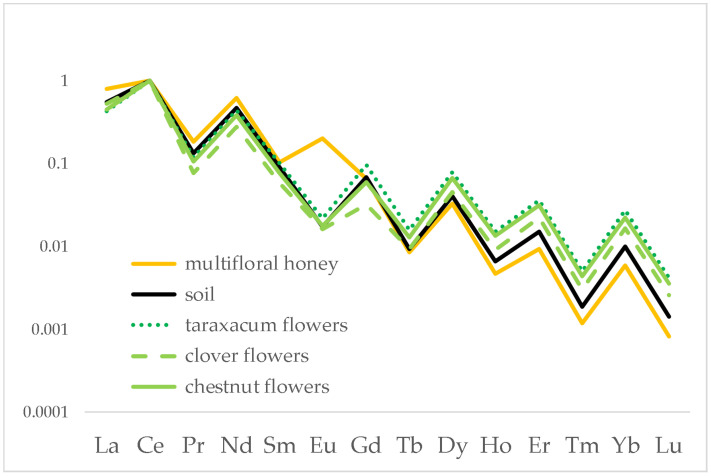
Distribution of lanthanides in multifloral honey from Robilante, obtained from chestnut, clover and taraxacum flowers. Data were normalised to Ce.

While the previous results were obtained the analysis of monofloral honey, in one case, we analysed a multifloral honey obtained from flowers of plants grown on the same area. Such a situation is reported in Figure 5. Here is shown the honey chain from Robilante in which honey was produced from flowers of chestnut, clover and taraxacum.

The chain appears to be more complicate to evaluate. Again, it is clear that a slight fractionation of the heavier lanthanides occurs when passing from soil (black line) to honey (red line). The flowers appear less correlated to the common soil in which they grew. It must be remembered, however, that the concentrations determined for the heavier lanthanides are close to the detection limits of the instrument used (tens or units of ng/L), so the uncertainty is higher.

### 3.4. Different Years

**Figure 6:**
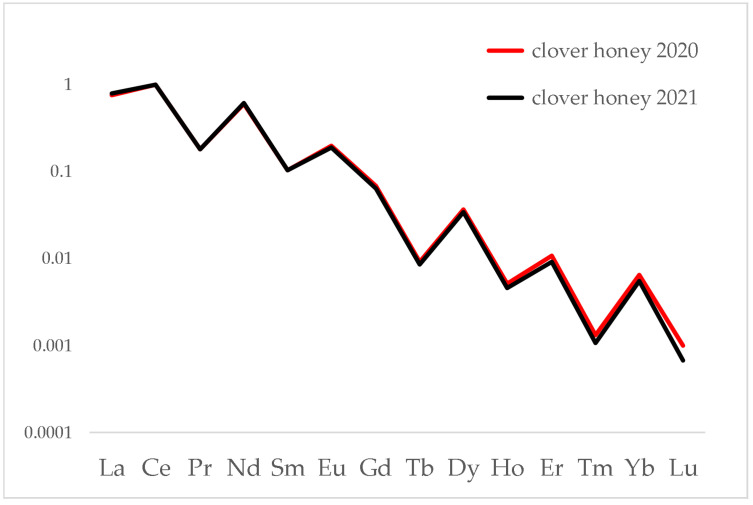
Distribution of lanthanides in clover honeys produced in 2020 and 2021 at the Colle del Lys site. Data were normalised to Ce.

It is interesting to compare the lanthanide distribution in honeys obtained from the same floral variety, the same territory but in different years. Figure 6 shows a comparison of two clover honeys produced at the Colle del Lys (Turin) site in 2020 and 2021.

The almost perfect overlapping of the distributions is apparent, despite any differences that may have arisen due to climatic conditions, growth of flowers or other factors. Again, a similar behaviour has been noted on other honey chains.

## 4. Discussion

**Figure 7:**
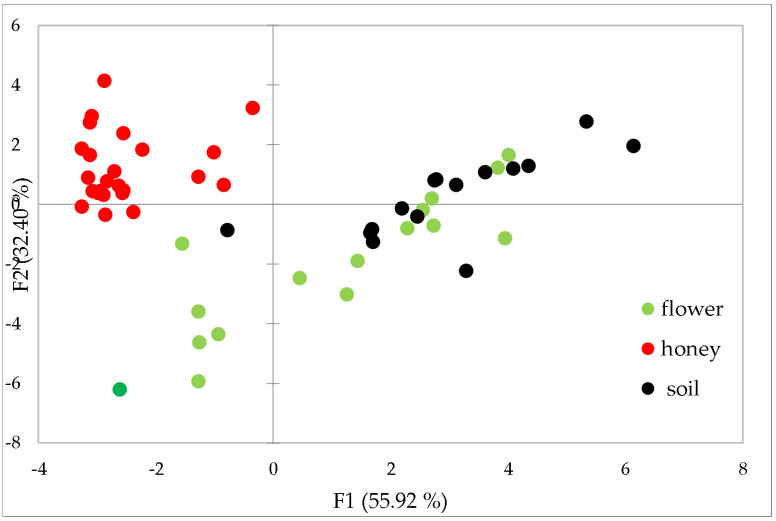
PC1 vs. PC2 plot of the data from ICP analysis of soils, flowers and honeys. The linden flower is marked in dark green.

The results obtained from the analysis of several different honey chains allow a common behaviour to be individuated: the typical fingerprint of soil, expressed by means of the lanthanide distribution, is passed almost unaltered to the flowers of the plants grown on that soil, as expected according to the scientific literature. After collection of the nectar from the flowers, bees transform it into honey and this passage seems to cause a slight fractionation on the heavy lanthanides, that is, from Dy to Lu, which content is lower in honeys than in soil/flowers. In fact, the behaviour shown in Figure 2 is common to all the honey chains analysed in the present study, confirming that a fractionation occurs for heavy lanthanides in the passage from soil to honey. In order to have a complete view of the dataset, a dimensionality reduction was applied by means of Principal Component Analysis using Ce normalised data. The resulting PC1 vs. PC2 biplot, accounting for 88.32% of explained variance, is shown in Figure 7.

All soil samples, except for one, cluster at positive values of PC1; flower samples cluster at negative values of PC2 but closer to soil samples, with the exception of linden flower (dark green in Figure 3), which is far from the soil cluster: this can be explained as the linden is a very tall tree (from 20 to 40 m) so that the relationship with soil could be less defined; honey samples, finally, cluster at negative values of PC1 and positive values of PC2. The separation of honey samples from soil + flower samples is mainly due, as expected, to the heavy lanthanides, which are higher in the soil + flower group.

We can hypothesise that the fractionation on heavy lanthanides is due to the metabolism of bees, also considering that the passage of the pollen involves the addition of enzymes, needed for the production of honey. A similar behaviour was noted in a study on the traceability of milk [30]: in that case, the role of the metabolism of cows was cited to justify a fractionation of the heavy lanthanides in the passage from soil to raw milk. One factor to consider could be the so-called *lanthanide contraction*, the well-known property of these elements according to which the size of atoms and ions decreases as the atomic number increases from lanthanum (La—atomic number 57) to lutetium (Lu—atomic number 71): this could influence the rate of excretion of lanthanides by bees, being the rate faster for heavier—and therefore smaller—lanthanides than for the lighter ones.

All the measurements carried out confirm this behaviour. Particularly significant is the fact that honey produced by the same bees from different botanical species growing in the same soil have a common behaviour, making apparent the fact that the botanical variety is not a factor inducing fractionation, while more significant is the contribution of soil (for lighter lanthanides) and of bees (for heavier lanthanides). The permanence of the behaviour in honeys produced in different years from the same botanical species and soil is also relevant. In the end, it seems as if the fingerprint of soil is well kept, although with the fractionation of heavy lanthanides, so that it could constitute a good basis for verifying the geographic provenance of honey. In fact, monofloral honeys produced in different countries could have different fingerprints.

## 5. Conclusions

The present study shows a comparison between soil, flowers and honey produced from these flowers using the lanthanides as chemical markers. The comparison was carried out by exploiting the distribution of lanthanides with data normalised to Ce in such a way to allow an easier comparison. This approach is different from the usual classification methods, in which absolute data are used to distinguish samples from different geographical origin or botanic species. In this sense, the present study can be considered as a pure traceability study.

The results allow the hypothesis that the honey chain is only partially traceable due to the fractionation of the heavy lanthanides. Nevertheless, further measurements on honeys produced in other geographic areas could allow it to be established whether the fractionation is relevant or not in linking soil, flowers and honey.

## Figures and Tables

**Table 1:** Analysis of certified material SRM 2586 (*Trace Elements in Soil Containing Lead from Paint*).

Element	Certified Values (mg/kg)	Uncertainty	Found (mg/kg)	r.s.d. (%)
Li	25 ^1^		23.3	3.0
Be	1.4 ^1^		0.82	5.4
Na	4680	730	2140	3.5
Mg	17,070	840	7294	1.2
Al	66,520	760	37,443	2.1
P	1001	77	1185	5.6
K	9760	180	3255	2.1
Ca	22,180	540	15,637	2.2
Ti	6050	660	5065	1.6
V	160 ^1^		122	1.3
Cr	301	45	141	3.4
Mn	1000	18	861	1.7
Fe	51,610	890	44,309	0.9
Co	35 ^1^		23	0.9
Ni	75 ^1^		45	2.3
Cu	81 ^1^		55	2.9
Zn	352	16	263	1.5
As	8.7	1.5	7.0	2.2
Se	0.6 ^1^		2.1	5.0
Y	21 ^1^		15	1.2
Rb	^2^		799	1.4
Sr	84.1	8.0	34.1	2.0
Nb	6 ^1^		1.1	2.3
Cd	2.71	0.54	2.28	0.8
Ba	413	18	286	1.3
La	29.7	4.8	23.9	1.7
Ce	58	8	47	1.3
Pr	7.3 ^1^		6.0	2.8
Nd	26.4	2.9	22.4	2.9
Sm	6.1 ^1^		4.5	3.3
Eu	1.5 ^1^		0.9	3.6
Gd	5.8 ^1^		4.0	2.1
Tb	0.9 ^1^		0.6	2.2
Dy	5.4 ^1^		3.2	1.6
Ho	1.1 ^1^		0.6	3.2
Er	3.3 ^1^		1.6	2.0
Tm	0.5 ^1^		0.2	1.3
Yb	2.64	0.51	1.34	1.3
Lu	^2^		0.2	3.9
Pb	432	17	365	3.3
Th	7 ^1^		5.7	5.5

^1^ Indicative value. ^2^ Not determined in SRM.

**Table 2:** Analysis of certified material SRM-1573A (*Tomato leaves*).

Element	Certified Values (mg/kg)	Uncertainty	Found (mg/kg)	r.s.d. (%)
Li	^2^		0.83	5.4
Be	^2^		0.036	9.4
Na	136	4	79	1.3
Mg	12,000 ^1^		10,150	2.1
Al	598	12	392	3.4
Si	^2^		145	5.2
P	2160	40	1969	6.3
S	9600 ^1^		10,478	4.7
K	27,000	500	15,463	1.4
Ca	50,500	900	59,548	2.4
Ti	^2^		5.73	10.0
V	0.835	0.010	0.222	3.3
Cr	1.99	0.06	0.61	4.0
Mn	246	8	245	3.2
Fe	368	7	224	2.6
Co	0.57	0.02	0.74	1.4
Ni	1.59	0.07	1.20	1.8
Cu	4.70	0.14	6.92	2.0
Zn	30.9	0.7	65.8	1.6
As	0.112	0.004	0.160	5.2
Se	0.054	0.003	0.139	31.9
Rb	14.89	0.27	22.35	2.3
Sr	85 ^1^		58	1.2
Y	^2^		0.94	1.0
Zr	^2^		0.33	1.6
Mo	0.46 ^1^		0.46	0.5
Cd	1.52	0.04	1.62	1.7
Ba	63 ^1^		56	2.0
La	2.3 ^1^		2.2	1.8
Ce	2 ^1^		2.0	0.8
Pr	^2^		0.37	2.1
Nd	^2^		1.2	3.5
Sm	0.19 ^1^		0.18	3.6
Eu	^2^		0.035	0.5
Gd	0.17 ^1^		0.15	4.4
Tb	^2^		0.021	2.6
Dy	^2^		0.097	1.3
Ho	^2^		0.020	2.9
Er	^2^		0.045	2.2
Tm	^2^		0.005	4.5
Yb	^2^		0.025	2.4
Lu	^2^		0.004	6.2
Pb	^2^		0.976	1.9
Th	0.12 ^1^		0.03	1.9
U	0.035 ^1^		0.022	5.0

^1^ Indicative value. ^2^ Not determined in SRM.

**Table 3:** Minimum, maximum and average values for soil, flower and honey samples analysed in this study.

Element	Soils		Flowers		Honeys	
	Min–Max	Average	Min–Max	Average	Min–Max	Average
Li	5.75–68.7	31.8	0.015–2.05	0.371	0.103–3.19	0.653
Be	0.181–1.07	0.626	0.000401–0.0518	0.009442	0.000026–0.001705	0.000266
B	124–348	133	^1^–23.8	1.71	^1^–3.29	
Na	102–2140	1344	6.76–272	64.6	0.979–43.5	15.7
Mg	9.85–14673	8158	874–5359	2918	0.093–11.7	2.69
Al	^2^	^2^	17.9–842	187	0.146–22.7	5.27
Si	^2^	^2^	9.16–549	148	0.340–14.9	2.90
P	514–5907	1531	1159–6429	3681	3.80–59.0	13.2
S	765–11,886	2727	800–4791	2858	1.58–53.2	8.01
K	969–7758	2587	4947–25,711	15,457	2.14–134	31.6
Ca	30.9–198,804	45,253	1572–27,171	12,575	0.266–44.4	23.1
Ti	134–6319	1987	0.449–26.6	6.34	0.006199–0.0808	0.0231
V	6.07–122	59.4	0.0131–1.63	0.263	0.001211–0.005467	0.002616
Cr	6.42–246	99.3	0.0738–2.75	0.773	0.000475–0.0112	0.004419
Mn	0.82–2430	730	11.1–181	62.4	0.000294–1.221	0.117
Fe	65.3–56,229	28,181	54.2–1341	249	0.0769–1.675	0.388
Co	1.04–25.9	13.4	0.0372–1.05	0.241	0.000115–0.002056	0.000531
Ni	3.87–143	69.5	0.964–26.8	8.81	0.001935–0.0325	0.007813
Cu	4.93–131	55.4	8.43–20.9	13.9	0.0101–0.312	0.0612
Zn	31.6–263	81.6	19.0–97.9	48.3	0.0138–0.195	0.0629
As	2.57–11.0	5.70	0.007002–1.29	0.176	0.000349–0.001607	0.000788
Se	0.645–3.99	1.74	0.005161–0.135	0.0529	0.000084–0.001401	0.000462
Rb	739–2325	1113	0.77–78.2	36.8	^1^–23.3	0.907
Sr	8.98–517	93.0	1.101–120	27.4	0.0752–0.424	0.191
Y	3.00–30.3	14.0	0.008261–2.26	0.255	0.000310–0.004471	0.001272
Zr	0.022424–10.1	0.635	0.002815–0.329	0.0852	0.000423–0.0149	0.002568
Nb	0.012129–4.27	1.09	^1^–^1^	^1^	0.000064–0.000417	0.000151
Mo	0.545–3.35	1.90	0.0414–11.9	3.17	0.001453–0.008853	0.004012
Cd	0.111–2.28	0.295	0.000012–0.148	0.0487	0.000008–0.000121	0.000039
Sb	0.174–2.05	0.538	0.000759–0.0755	0.0390	0.000007–0.000142	0.000029
Ba	0.75–356	155	0.969–64.9	16.0	0.0075–0.733	0.410
La	5.90–36.6	19.0	0.008276–1.36	0.257	0.002922–0.0196	0.009173
Ce	12.7–64.8	36.8	0.0188–1.73	0.383	0.003596–0.0317	0.0107
Pr	1.51–9.18	4.70	0.001485–0.214	0.0480	0.000645–0.005398	0.002205
Nd	6.02–34.3	17.5	0.005083–0.795	0.172	0.002079–0.0190	0.007551
Sm	1.45–8.13	3.75	0.000921–0.187	0.0352	0.000334–0.003780	0.001317
Eu	0.166–2.02	0.772	0.000431–0.0402	0.008415	0.000771–0.003210	0.001644
Gd	1.32–7.70	3.44	0.001285–0.198	0.0370	0.000203–0.002514	0.000842
Tb	0.180–1.20	0.546	0.000200–0.0297	0.005955	0.000027–0.000365	0.000114
Dy	0.785–6.22	2.88	0.001274–0.159	0.0305	0.000105–0.001568	0.000458
Ho	0.130–1.30	0.581	0.000269–0.0380	0.006381	0.000015–0.000222	0.000064
Er	0.295–3.39	1.50	0.000675–0.0903	0.0154	0.000029–0.000470	0.000131
Tm	0.0368–0.513	0.219	0.000098–0.0106	0.002015	0.000004–0.000061	0.000016
Yb	0.194–2.95	1.24	0.000571–0.0514	0.0103	0.000019–0.000335	0.000085
Lu	0.0277–0.476	0.193	0.000091–0.007972	0.001665	0.000002–0.000050	0.000012
W	0.0216–1.49	0.357	^1^–0.418	^1^	0.001962–0.0952	0.0204
Tl	0.0855–0.685	0.276	0.000541–0.0799	0.0129	0.000002–0.000730	0.000061
Pb	12.3–365	39.9	0.0794–3.88	0.646	0.001545–0.0206	0.004516
Th	1.18–12.2	5.94	0.001084–0.118	0.0196	0.000221–0.004136	0.000883
U	0.373–4.26	1.58	0.000972–0.0541	0.0103	0.000047–0.001480	0.000218

^1^ Below detection limit. ^2^ Not determined in these samples.

## Data Availability

The data presented in this study are available on request from the corresponding author.

## References

[1] Zappi A., Melucci D., Scaramagli S., Zelano A., Marcazzan G.L. (2018). Botanical Traceability of Unifloral Honeys by Chemometrics Based on Head-Space Gas Chromatography. Eur. Food Res. Technol..

[2] (2019). Codex Alimentarius Commission Standard for Honey CXS 12-1981.

[3] EU Council (2002). Council Directive 2001/110/EC of 20 December 2001 Relating to Honey. Off. J. Eur. Communities.

[4] Moore J.C., Spink J., Lipp M. (2012). Development and Application of a Database of Food Ingredient Fraud and Economically Motivated Adulteration from 1980 to 2010. J. Food Sci..

[5] Pohl P. (2009). Determination of Metal Content in Honey by Atomic Absorption and Emission Spectrometries. TrAC—Trends Anal. Chem..

[6] Magdas D.A., Guyon F., Puscas R., Vigouroux A., Gaillard L., Dehelean A., Feher I., Cristea G. (2021). Applications of Emerging Stable Isotopes and Elemental Markers for Geographical and Varietal Recognition of Romanian and French Honeys. Food Chem..

[7] Sabatini A.G., Bortolotti L., Marcazzan G.L. (2007). Conoscere Il Miele.

[8] Lestari L.A., Triyana K., Hanifah A.K., Wildiana R.A. (2021). The Use of Electronic Tongue (e-Tongue) as a Simple and Rapid Method for Honey Authentication. Food Res..

[9] Martin P. (2005). Importance of Melissopalynology for Beekeeping and Trade. Bee World.

[10] Corvucci F., Nobili L., Melucci D., Grillenzoni F.V. (2015). The Discrimination of Honey Origin Using Melissopalynology and Raman Spectroscopy Techniques Coupled with Multivariate Analysis. Food Chem..

[11] Davies A.M.C., Radovic B., Fearn T., Anklam E. (2017). A Preliminary Study on the Characterisation of Honey by near Infrared Spectroscopy. J. Near Infrared Spectrosc..

[12] Tan S.H., Pui L.P., Solihin M.I., Keat K.S., Lim W.H., Ang C.K. (2021). Physicochemical Analysis and Adulteration Detection in Malaysia Stingless Bee Honey Using a Handheld Near-infrared Spectrometer. J. Food Process. Preserv..

[13] Berghian-Grosan C., Hategan A.R., David M., Magdas D.A. (2023). Untargeted Metabolomic Analysis of Honey Mixtures: Discrimination Opportunities Based on ATR-FTIR Data and Machine Learning Algorithms. Microchem. J..

[14] Sahlan M., Ahlam N.A.L., Agus A., Sabir A., Pratami D.K. (2022). Identification and Authentication of Honey Using Chemometric Analysis Based on ATR-FTIR and Raman Spectroscopy. Int. J. Appl. Pharm..

[15] Sotiropoulou N.S., Xagoraris M., Revelou P.K., Kaparakou E., Kanakis C., Pappas C., Tarantilis P. (2021). The Use of SPME-GC-MS IR and Raman Techniques for Botanical and Geographical Authentication and Detection of Adulteration of Honey. Foods.

[16] Escriche I., Kadar M., Domenech E., Gil-Sánchez L. (2012). A Potentiometric Electronic Tongue for the Discrimination of Honey According to the Botanical Origin. Comparison with Traditional Methodologies: Physicochemical Parameters and Volatile Profile. J. Food Eng..

[17] Karabagias I.K., Badeka A.V., Kontakos S., Karabournioti S., Kontominas M.G. (2014). Botanical Discrimination of Greek Unifloral Honeys with Physico-Chemical and Chemometric Analyses. Food Chem..

[18] Ye H., Yang J., Xiao G., Zhao Y., Li Z., Bai W., Zeng X., Dong H. (2023). A Comprehensive Overview of Emerging Techniques and Chemometrics for Authenticity and Traceability of Animal-Derived Food. Food Chem..

[19] Danieli P.P., Lazzari F. (2022). Honey Traceability and Authenticity. Review of Current Methods Most Used to Face This Problem. J. Apic. Sci..

[20] Zhang G., Abdulla W. (2022). On Honey Authentication and Adulterant Detection Techniques. Food Control.

[21] Siddiqui A.J., Musharraf S.G., Choudhary M.I., Rahman A. (2017). Application of Analytical Methods in Authentication and Adulteration of Honey. Food Chem..

[22] Tsagkaris A.S., Koulis G.A., Danezis G.P., Martakos I., Dasenaki M., Georgiou C.A., Thomaidis N.S. (2021). Honey Authenticity: Analytical Techniques, State of the Art and Challenges. RSC Adv..

[23] Aceto M., Espiñeira M., Santaclara F.J. (2016). The Use of ICP-MS in Food Traceability. Advances in Food Traceability Techniques and Technologies.

[24] Mazarakioti E.C., Zotos A., Thomatou A.-A., Kontogeorgos A., Patakas A., Ladavos A. (2022). Inductively Coupled Plasma-Mass Spectrometry (ICP-MS), a Useful Tool in Authenticity of Agricultural Products’ and Foods’ Origin. Foods.

[25] Drivelos S.A., Danezis G.P., Halagarda M., Popek S., Georgiou C.A. (2021). Geographical Origin and Botanical Type Honey Authentication through Elemental Metabolomics via Chemometrics. Food Chem..

[26] Voica C., Iordache A.M., Ionete R.E. (2020). Multielemental Characterization of Honey Using Inductively Coupled Plasma Mass Spectrometry Fused with Chemometrics. J. Mass Spectrom..

[27] Weilert T.M., Ray C.L., Gawenis J.A., Brockman J.D. (2022). Neutron Activation Analysis and ICP-MS for Provenance of Honey Collected from American Midwest Region. J. Radioanal. Nucl. Chem..

[28] Oddone M., Aceto M., Baldizzone M., Musso D., Osella D. (2009). Authentication and Traceability Study of Hazelnuts from Piedmont, Italy. J. Agric. Food Chem..

[29] Aceto M., Calà E., Musso D., Regalli N., Oddone M. (2019). A Preliminary Study on the Authentication and Traceability of Extra Virgin Olive Oil Made from Taggiasca Olives by Means of Trace and Ultra-Trace Elements Distribution. Food Chem..

[30] Aceto M., Musso D., Calà E., Arieri F., Oddone M. (2017). Role of Lanthanides in the Traceability of the Milk Production Chain. J. Agric. Food Chem..

[31] Aceto M., Gulino F., Calà E., Robotti E., Petrozziello M., Tsolakis C., Cassino C. (2020). Authentication and Traceability Study on Barbera d’asti and Nizza Docg Wines: The Role of Trace-and Ultra-Trace Elements. Beverages.

[32] Aceto M., Robotti E., Oddone M., Baldizzone M., Bonifacino G., Bezzo G., Di Stefano R., Gosetti F., Mazzucco E., Manfredi M. (2013). A Traceability Study on the Moscato Wine Chain. Food Chem..

[33] Somanathan H., Saryan P., Balamurali G.S. (2019). Foraging Strategies and Physiological Adaptations in Large Carpenter Bees. J. Comp. Physiol. A.

[34] Calà E., Fracchia A., Robotti E., Gulino F., Gullo F., Oddone M., Massacane M., Cordone G., Aceto M. (2022). On the Traceability of the Hazelnut Production Chain by Means of Trace Elements. Molecules.

[35] Oddo G. (1914). Die Molekularstruktur Der Radioaktiven Atome. Z. Anorg. Chem..

[36] Brown P.H., Rathjen A.H., Graham R.D., Tribe D.E., Gschneider K.A., Eyring L. (1990). Rare Earth Elements in Biological Systems. Handbook on the Physics and Chemistry of Rare Earths.

[37] Tyler G. (2004). Rare Earth Elements in Soil and Plant Systems—A Review. Plant Soil.

[38] Liang T., Ding S., Song W., Chong Z., Zhang C., Li H. (2008). A Review of Fractionations of Rare Earth Elements in Plants. J. Rare Earths.

